# Supplementary material for: Are carbon emissions trading and green financial instruments synergistic? -Comprehensive quantitative research based on content analysis
Source: PLoS One. 2024 Mar 7;19(3):e0298601. doi: 10.1371/journal.pone.0298601 (PMC10919601; doi:10.1371/journal.pone.0298601)
Supplement: S1 Data — (ZIP) [file pone.0298601.s001.zip › Data/地级市Z_F工作报告.zip!/╦─┤¿/┤∩╓▌╩╨/└┤╫╘╣1⁄2╓┌║┼í╛┬φ┐╦╩2╛▌═°í┐_2019.docx]

各位代表：

我代表市人民政府，向大会报告工作，请予审议，并请市政协委员提出意见。

一、2018年工作回顾

2018年，在省委省政府和市委的坚强领导下，我们坚持以习近平新时代中国特色社会主义思想为指导，认真落实省委十一届三次全会和市委四届六次全会决策部署，以“八个年”活动[1]为抓手，科学谋划、精准施策、攻坚克难，圆满完成市四届人大三次会议确定的各项目标任务。全年实现地区生产总值1690.17亿元、增长8.3%，地方一般公共预算收入101亿元、增长10.05%，全社会固定资产投资增长12.3%，社会消费品零售总额增长12.3%，城镇、农村居民人均可支配收入分别增长8.8%和9.4%，各项经济指标增速均高于年初预期、高于全省平均水平，全市经济在“十三五”以来止滑回升、巩固提高的基础上全面步入健康向好发展轨道，为决胜同步全面小康、争创全省经济副中心奠定了坚实基础。

（一）聚力稳增长促转型，经济发展持续向好。坚持一手抓存量改造提升、一手抓增量培育壮大，全市经济实现速度质量“双提升”。结构调整步伐加快，服务业占GDP的比重同比提高0.6个百分点，三次产业结构调整为19.3:35.7:45，传统工业占规上工业的比重降至58%。经济质量明显提高，财政收入增速持续高于GDP增速，税收占一般公共预算收入比重同比提高4.6个百分点，单位GDP能耗下降4%。项目建设强力推进，34个重大项目和230个重点项目带动投资完成1400.4亿元，招商签约项目179个、引进到位资金833亿元。工业经济巩固提升，新增规上企业87户，规上工业增加值增长10.6%、高于全省2.3个百分点，利润增长94.8%，玄武岩纤维产业园、微玻纤产业园、川渝合作示范园等加快建设，达州经开区扩区调位、市第二工业园区建设工作正式启动，大竹、宣汉等5个县区工业园区成功创建为省级开发区。建筑产业持续壮大，三年跨越3个“百亿”台阶，实现总产值515.6亿元、增长28.7%，市建筑建材产业园开工建设，三一筑工装配式建筑签约落地。商贸物流业迅猛发展，秦巴物流园区、双龙公铁物流园等重点项目加快建设，复兴农副产品批发市场、升华广场等建成投运，第八届秦巴商品交易会成功举办，我市成功列入国家物流枢纽承载城市。文化旅游业加快发展，旅游总收入、人次分别增长22.1%和26.8%，按照国家5A级景区标准打造的巴山大峡谷一期隆重开园，全市集中推出的16个重点乡村旅游景点提档升级，“巴山夜雨·水墨达州”品牌影响逐步扩大。金融业稳步发展，存款余额2927亿元，贷款余额1503亿元，存贷比同比提高5.7个百分点。现代农业加快发展，粮食产量稳中有增，新建现代农业产业基地13万亩，农产品加工和贸易收入260亿元、增长12%，开江“稻田+”融合园区作为典型案例在全省推广。科技创新深入推进，建成各类创新平台12家，新增国家高新技术企业8家，科技对经济增长的贡献率同比提高3个百分点。县域经济支撑有力，5个县区GDP超过200亿元，其中，2个县区GDP超过300亿元，4个县区地方一般公共预算收入超过10亿元。

（二）聚力打基础强管理，城乡面貌显著改观。基础设施建设取得重大进展，成南达万高铁被列为省上重点工程，西达渝高铁、达州高铁站、达万铁路扩能改造、固军水库等项目前期工作扎实推进，达州机场迁建、营达和巴万高速、达开和达宣快速通道、“1+6”大中型水库[2]等项目加快实施。中心城区建设成效显著，罗江、中坝等4座大桥开工建设，环城路二期、快速通道西段、南城III号干道等建成通车，金南大桥、凤凰山隧道、火烽山隧道、大尖子隧道等控制性工程运行顺畅，新建停车泊位2万个，困扰主城区多年的交通拥堵得到极大改善；马踏洞、莲花湖、三里坪、长田坝等新区开发扎实推进，建成区面积突破100平方公里、人口达105万人，“双百”城市[3]如期建成；塔沱、火车站、川鼓等片区棚改加快实施，应急备用水源开工建设，莲花湖生态湿地公园、城市运动公园顺利推进，凤凰山游步道、紫荆花公园等建成开放，大北街商业示范街实现提档升级，老城区燃气管网改造、道路修补改造全面完成，城市功能品质不断完善提升；“城市综合管理年”活动有序开展，全国文明城市和国家森林城市、园林城市、卫生城市创建工作同步推进，主城区环卫市场化清扫保洁制度性改革实现全覆盖，保洁质量显著提升，智能交通二期基本建成，公共交通能力切实加强，城市管理水平明显提高。5个县城扩容提质，新增建成区面积8平方公里，大竹荣获“全国文明城市”提名城市，宣汉纳入全省“城市双修”试点城市。百镇建设深入开展，新增省级试点镇培育对象17个，达川百节、宣汉胡家成功创建省级特色镇。乡村振兴开局良好，通川纳入全省乡村振兴规划试点县，达川万家、宣汉渡口纳入全省试点乡镇，建成美丽宜居乡村500个，创建中国美丽休闲乡村1个。

（三）聚力抓重点补短板，三大攻坚战成效显著。脱贫攻坚决战告捷，15.17万人摆脱贫困，319个村脱贫退出，6.78万人易地搬迁，4个省级贫困县达到“摘帽”条件正待省上验收，全市贫困发生率从上年的3.67%降至0.88%，大竹易地扶贫搬迁“双靠近三融合”[4]经验获国务院通报表扬，渠县扶贫移民局局长张渠伟获全国脱贫攻坚贡献奖，达州和渠县、大竹、通川分别获全省脱贫攻坚工作先进市、县。环境治理攻坚成效明显，中央和省环保督察及“回头看”反馈问题整改扎实推进，环保产业园和主城区生活垃圾发电厂迁建开工建设，市第二污水处理厂和55个城镇污水处理厂建成投运，城市黑臭水体、道路扬尘、达钢等重点企业污染治理取得阶段性成果，主城区PM2.5、PM10[5]平均浓度分别下降5.2和2.5个百分点，空气质量优良天数达295天；河（湖）长制全面落实，出境断面水质全部达到国家和省上标准；危及主城区生态屏障安全的松材线虫病防治强力推进。重大风险防范化解有力有效，政府债务保持在可控范围内，互联网金融风险专项整治扎实开展，地方法人银行机构不良贷款率下降1.7个百分点。

（四）聚力增动能添活力，改革开放成果丰硕。对接落实省上专项改革50项，部署推动重点领域改革20项。市本级制度性改革取得突破性进展，坚持“强市活区”理念构建新的市区管理体制，中心城区在规划、建设、国土管理等领域的责权利关系得到调整理顺；城管执法整合下沉，执法成效明显提升；市级机关事业单位绩效改革不断深化，干部职工工作积极性得到有效激发。国资国企改革扎实推进，34户市属破产国企改革成果进一步巩固；市级国有投资平台公司“平台转型”不断做实，在财政不投入的情况下基本完成了10类26户国企的基础改革。财政管理改革深入推进，市级机关事业单位经营性资产实现收入统一管理，市本级资金筹措使用统筹机制不断深化完善。供给侧结构性改革成效显著，落实减税降费政策减免税收36亿元；采取政府补贴、供需协商等多种措施推进水、电、气等要素降成本，全市工业园区符合条件的企业用电用水用气价格均控制在0.55元/度、1.86元/吨和1.88元/立方米以内，远低于全省平均水平。开放合作成效明显，争取多年的“达州海关”正式成立，与川东北经济区兄弟市和成都市在联动发展、协同发展方面的合作不断深化，与浙江舟山的扶贫、劳务、产业协作全面开展，与重庆万州在交通、物流等领域的合作不断加强，与央企省企、科研院所在玄武岩纤维开发等项目合作不断拓展，与清华大学、四川大学、成都中医药大学等高等院校在人才培养引进、办医办学等方面的合作成效显著，与农发行、国开行等金融机构在易地扶贫搬迁、城市棚户区改造等方面的合作不断扩大，与省农担公司合作启动全市农业担保工作。

（五）聚力惠民生促和谐，人民福祉不断改善。各项民生支出296亿元，占一般公共预算支出比重连续7年超过70%，九项民生工程、18件民生实事如期完成。城镇新增就业4.3万人。完成城市棚户区改造1.5万套，农村危房和土坯房改造7.4万户。全民参保计划深入实施，五项社会保险参保覆盖1008万人次。异地就医直接结算逐步推开。养老条件不断改善，新增床位914张。教育事业加快发展，主城区学前教育“三年攻坚行动计划”已经拟定；新改扩建中小学校117所，7个县市区义务教育基本均衡发展全部达标；市本级和7个县市区集中力量办强一所重点公办高中成效初显，成都天立、绵阳东辰、川师大附属实验学校等落地建设；职业教育加快发展，主城区市级中职学校整合入园稳步推进，达州职业技术学院加快搬迁入园，达州中医药职业学院加快建设，达州技师学院成功申办；无偿划拨土地308亩，支持四川文理学院提质扩容。健康达州行动深入开展，与华西妇女儿童医院合作办医加快建设，与华西医院、省骨科医院、奥美德国际医院等合作办医深入洽谈，市中心医院、市中西医结合医院和县级人民医院软硬件建设得到加强，乡村医疗机构达标建设成效明显。食品药品监管执法力度加大，“地沟油”整治持续推进，主城区餐厨垃圾初步实现无害化处理，非洲猪瘟防控有力有效。安全生产责任制严格落实，事故起数、死亡人数同比分别下降38.5%和36.5%。平安达州建设深入推进，扫黑除恶专项斗争持续开展，国家禁毒重点县全面摘帽，滨江明珠、洲河花园等信访积案难案得到有效解决，通川塔沱火灾、达川街面塌陷等突发事件得到妥善处置，公共安全持续巩固，退役军人服务管理工作成效显著，社会大局保持和谐稳定。

（六）聚力转作风提效能，自身建设切实加强。全面加强思想政治建设，政府系统干部“四个意识”[6]持续增强、“四个自信”[7]更加坚定、“两个维护”[8]更加自觉。认真开展“大学习、大讨论、大调研”，深入研究谋划事关达州长远发展的重大问题，政府工作思路举措更加科学务实。坚持市政府常务会学习法律法规和中、省重要文件，出台了我市第一部政府规章，提请审议地方性法规草案2件，法治政府建设取得新成效。定期向市人大常委会报告工作、向市政协通报情况，自觉接受市人大、市政协监督，办理人大代表建议277件、政协提案361件。出台了进一步改进作风提升工作实效的3项规定，政府系统文风会风明显改进，调研考察实效明显提高。借鉴全国先进经验，制定出台了全省前置最少、流程最优、效率最高的项目审批服务制度、“最多跑一次”等4项重大行政审批和公共服务改革方案。“互联网+政务服务”持续深化，全流程电子化交易平台功能不断完善，异地远程评标走在全省前列，政务服务事项网上可办率达79%。政府机构改革有序推进。强化审计监督和权力运行监督，大力支持纪委监委查处违纪违法案件，政府系统党风廉政建设切实加强。

与此同时，我们统筹兼顾，积极支持全市各条战线开展工作，国防动员、国家安全、人民防空、民兵预备役、优抚安置等工作切实加强，妇女、儿童、老龄、残疾人、慈善、红十字会等事业全面发展，民族、宗教、统计、气象、保密、档案、地方志等工作取得新的成绩。

各位代表！过去一年的成绩来之不易。这是省委省政府和市委坚强领导的结果，是市人大和市政协大力支持的结果，是全市广大干部群众苦干实干的结果。在此，我代表市人民政府，向全市人民，向各位人大代表、政协委员，向各民主党派、工商联、无党派人士和人民团体，向驻达解放军指战员、武警官兵和政法干警，向所有关心、支持和参与达州建设发展的社会各界朋友，表示衷心的感谢！

在看到成绩的同时，我们也清醒地认识到，全市经济社会发展还面临不少困难和挑战，政府工作还存在一些不足，主要是：经济总量不够大、结构不够优、质量不够高，持续向好发展的基础还不牢；城乡基础设施建设短板多、欠账大、任务重；教育医疗的总量和质量与广大人民群众的期盼还有较大差距；改革创新、开放合作的强度和力度还不适应加快发展需要；土地、资金、环境容量等要素制约还比较突出；干部队伍的能力素质、政务服务的质量效率还有待进一步提高。对此，我们一定高度重视，采取有力措施持之以恒推动解决。

二、2019年工作总体安排

2019年，是新中国成立70周年，是全面建成小康的关键之年，也是达州建市20周年，做好各方面工作至关重要。今年政府工作的总体要求是：坚持以习近平新时代中国特色社会主义思想为指导，认真学习党的十九大精神和习近平总书记对四川工作系列重要指示精神，深入贯彻中央、省委省政府和市委的各项决策部署，认真践行“六个稳”[9]要求和“八字”方针[10]，贯彻落实省委十一届三次、四次全会精神和市委四届六次、七次全会精神以及中省市经济工作会议精神，以争创全省经济副中心规划为蓝图，继续深入开展“八个年”活动，持续打好“三大攻坚战”，进一步优化营商环境，提振市场信心，扩大有效投资，壮大实体经济，发展现代产业，统筹抓好稳增长、促改革、调结构、惠民生、防风险工作，强化专题专班机制，强力推进改革发展各项工作，确保经济持续健康发展、社会和谐稳定，为全面建成小康社会和奋力争创全省经济副中心奠定坚实基础。

今年经济社会发展的主要预期目标是：地区生产总值增长8%以上，全社会固定资产投资增长10%，社会消费品零售总额增长10.5%，地方一般公共预算收入增长8%，城镇、农村居民人均可支配收入分别增长8.6%和9.3%，城镇登记失业率控制在4.2%以内，居民消费价格涨幅控制在3%左右，完成省上下达的环境保护和节能减排目标任务。

各位代表！虽然当前宏观经济形势“稳中有变”甚至“变中有忧”，但中央判断我国发展仍处于并将长期处于重要战略机遇期没有改变。达州通过近几年的调整、巩固和提升，全市经济社会发展处于持续向好的趋势也没有改变。提出上述目标，综合考虑了外部宏观形势和我市发展条件，兼顾了发展需要和可能。做好今年的政府工作，实现上述目标，我们必须坚定信心并坚持以下原则。

——必须坚持稳中求进。稳中求进是做好经济工作的基本遵循。面对“稳中有变”的宏观形势，我们必须着力“稳”、巩固“稳”，以“稳”求“进”，继续把稳增长作为主要任务，精准施策稳产业、稳投资、稳消费，全力以赴稳金融、稳就业、稳预期，为加快发展创造环境、赢得空间。同时，要实现省委对达州新的定位要求，在确保质量的前提下必须致力“进”、加快“进”，在实际工作中我们将按照高于预期目标来安排布置今年的各项工作，以较高增速进行追赶，以积极姿态争创全省经济副中心。

——必须坚持高质量发展。高质量发展是党的十九大作出的重大决策。我们必须全面贯彻“巩固、增强、提升、畅通”八字方针，将巩固“三去一降一补”[11]成果、增强微观主体活力、提升产业链水平、畅通经济循环贯穿到经济工作全领域全过程，以构建现代经济体系支撑发展行稳致远。必须大力实施创新驱动战略，加快转变经济发展方式、优化经济结构、转换增长动力，聚力发展“6+3”[12]重点产业，确保在新一轮竞争中赢得新优势。

——必须坚持补齐短板。补齐短板是加快发展和缩小与发达地区差距的根本要求。我们将抢抓国家政策窗口期和省上开展基础设施等领域补短板三年行动的重大机遇，千方百计争取国家和省上重大项目布局，加快推进一批重大交通、水利、能源、生态环保、市政基础、城乡公共服务设施等建设，尽快补齐发展短板，推动城乡区域协调发展，加快缩小与发达地区的差距。

——必须坚持改革开放。改革开放是欠发达地区追赶跨越的制胜法宝。我市地处内陆山区，要实现加快发展，必须以更大的强度和力度深化改革、扩大开放。我们将坚持向改革要速度、要效益，研究出台更多体现达州特色、解决达州问题的改革举措，最大限度地激发内生动力。牢固树立开明开放发展理念，着力营造一流的营商环境，切实加强招商引资和对外合作，努力争取更多资金、技术、人才、资源和市场，借势借力推进发展。

——必须坚持发展为民。发展为民是政府工作的基本价值取向。我们将继续聚焦人民群众所想所盼所急，采取超常举措，切实加大工作力度和投入力度，着力解决好看病难、入学难、就业难等群众反映强烈的突出问题。始终坚持以人民为中心的发展思想，坚决办好承诺的民生实事，坚决打赢精准脱贫和生态环保攻坚战，确保民生支出只增不减，让人民群众得到更多实惠。

三、2019年重点任务

面对今年艰巨繁重的改革发展稳定任务，我们将突出重点、统筹兼顾，着力抓好以下九个方面的工作。

（一）全力推动经济高质量发展。坚持把加快发展作为第一要务，千方百计做大经济总量，全力以赴做优经济质量。

持续扩大有效投资。深入开展“项目攻坚突破年”活动，力争在项目储备、项目争取、项目建设等方面取得新的重大突破，确保全社会固定资产投资完成1530亿元以上。密切跟踪国家宏观经济政策走向，抢抓机遇，持续开展项目谋划，千方百计向上争取，力争全年储备重大项目100个、纳入省重点项目投资计划30个以上。继续实施重大项目带动战略，全力推进45个重大项目和235个重点项目建设，力争重点项目新开工56个、竣工54个。加强政务服务，抓好要素保障，充分发挥政府投资撬动作用和国有企业投资引领作用，鼓励更多社会资本参与达州建设，力争民间投资、产业投资分别增长10%以上。大力开展“招商引资落地年”活动，围绕“6+3”重点产业，瞄准世界500强、中国500强、民营500强企业招大引强，力争引进到位资金900亿元以上，引进500强企业10家以上。

加快建设工业强市和建筑业大市。大力开展“工业提质增效年”活动，持续推进六大产业集群发展，确保净增规上企业50户以上，规上工业增加值增长9%以上。着力调整和优化工业战略布局，推进达州经开区扩区调位，按照高新产业园区标准加快扩建现有经开区，开工建设市第二工业园和达钢搬迁项目，构建“一区两园”产业新格局，筑牢全市工业发展主体支撑；加快推进国家天然气综合开发利用示范区建设，继续抓好玄武岩纤维产业园、微玻纤产业园、川渝合作示范园建设，开工建设普光锂钾综合开发产业园；支持各县市区优化产业园区布局，发展具有比较优势的主导产业。切实加强园区基础设施建设，新建标准厂房40万平方米，新增道路20公里、土地储备3500亩，全面建成园区污水处理设施。加快产业链条构建和提升，突出抓好以“双纤+N”为重点的新材料产业链，以“双车+N”为重点的汽车机械产业链，以勘探开发、储气、管输、燃气发电、精细化工为重点的天然气全产业链，以达钢集团为重点的金属及非金属建材产业链发展，深入谋划和推进电子信息、磷硫化工、纺织服装、农产品加工、锂钾、中医药大健康、通用直升机应用等产业建链延链补链发展。加快推进川东北电信灾备中心建设及大数据产业园规划工作。大力发展现代建筑建材产业，加快建设市建筑建材产业园，推动三一筑工装配式建筑尽早开工，争取中国铁建落户达州发展高铁建设材料；支持本地建筑企业提档升级和品牌创建，鼓励市外建筑企业迁入达州，新增建筑资质企业60家，建筑业总产值增长20%以上。

大力发展现代服务业。狠抓服务业提档升级措施落地见效，确保服务业增加值增长9.7%以上。大力推进文化旅游业发展，加强“巴山夜雨·水墨达州”旅游品牌宣传，以巴山大峡谷为旗舰，全力推进北部片区“一山一河一谷”[13]、中心城区“两山两湖两岸”[14]综合开发，继续提档升级20个重点乡村旅游景点，加快以城坝和罗家坝为重点的巴文化遗址考古发掘，建成巴文化主题展陈馆和非遗陈列馆，鼓励有条件的县市区争创“天府旅游名县”，力争旅游总收入250亿元以上。着力发展口岸物流，加快推进秦巴物流园区、双龙公铁物流园等重点项目建设，抓好海关出口监管仓、进口保税仓和物流信息化建设，开展与浙江舟山的物流对接，争取广西防城港冷链物流专列延伸至达州。繁荣发展商贸流通业，持续推进商贸流通信息化、标准化、集约化改造，切实解决商贸业现代化问题；加快推进通川复兴、达川杨柳等商贸集聚区建设，着力打造完善仁和春天、红星国际、通川大北街、达川升华核心商圈等城市综合体和特色商业街，扶持发展一批行业龙头企业。大力发展电子商务，重点抓好电商集聚区和示范县建设，加快引进培育一批大型电商平台和大型电商企业。加强市场调控和监管，确保房地产市场平稳健康发展。抓好健康养老产业发展，支持各地结合生态旅游建设康养基地，推动医养融合发展。

做大做强县域经济。县域经济是我市做大经济总量和争创全省经济副中心的基础支撑。强化分类指导和政策支持，鼓励各县市区发挥优势、竞相跨越，争创全省县域经济发展强县和生态示范标杆县，力争4个县区GDP超250亿元，其中3个县区GDP超300亿元、3个县区财政收入超15亿元。突出主城区引领骨干作用，支持通川区、达川区和达州经开区错位发展、互补发展、联动发展，依托主城区交通、区位、产业等优势，大力发展新兴制造业、现代建筑业和服务业，引领全市经济高质量发展，带动县域经济加快发展。支持万源、开江特色发展，依托与陕西和重庆交界、生态环境较好等优势，致力发展区域性商贸物流、农产品加工、生态旅游、生物医疗、健康养老、特色种养业。支持宣汉、大竹、渠县全面发展，依托人口较多、经济体量较大等优势，突出发展制造业、建筑业、商贸物流业、金融业和旅游业。

发展壮大民营经济。全面落实国家、省、市促进民营经济发展政策措施，确保民营经济增加值占GDP的比重达61%以上。帮助指导民营企业建立现代企业制度，完善公司法人治理结构。构建亲清新型政商关系，瞄准民营企业发展瓶颈制约，全面推行代办服务，帮助解决融资难、用地难、手续办理难等实际困难。全面实施市场准入负面清单制度，依法保护企业家财产权、创新权和自主经营权。支持民营企业坚守主业实业、转型升级、创新发展，提升市场竞争力。以更加优惠的政策鼓励“个转企”“小升规”，培育壮大“四上一新”[15]骨干企业。引导民营企业坚守正道、依法经营、回馈社会，让诚信守法、扶贫济困成为达州民企的最好名片。

（二）聚力建设现代综合交通枢纽。根据省委对达州的新定位和人流、物流、产业布局的新变化，优化完善交通规划布局，加强铁公水空和信息化建设，着力构建外联内畅的现代交通运输体系，加快建设四川东出北上综合交通枢纽。

全力推进铁路枢纽建设。全力支持成南达万高铁开工建设，扎实做好西渝高铁、达州高铁南站、达万铁路扩能改造、达州至广元城际铁路前期工作，开展绵阳-阆中-达州铁路规划研究，规划建设火车货站二期、石梯铁路专用线，加快构建区域铁路枢纽。

着力推进公路路网建设。建成营达高速，加快建设巴万高速，力争开工市绕城高速西段，加快达万直达、镇巴-达州-广安、城口-宣汉-大竹-邻水、通江-宣汉-开州、开江-梁平、大竹-垫江、城口-万源高速前期工作，开展绵阳-达州高速规划研究。建成机场大道、达宣快速通道，加快建设达开快速通道，开工建设G210线达州市过境段三期工程、营达高速涌兴至市第二工业园区快速通道，积极做好达州-石桥、宣汉-南坝、达州-渠县、大竹-百节机场和巴山大峡谷-龙潭河-八台山快速通道前期工作。加强农村公路建设，消除“断头路”，打通“最后一公里”。

大力推进航空水运建设。加快达州机场迁建，确保建成机场跑道和航站区主体工程。力争新开通航线1条以上。科学谋划通用航空规划布局，争取在达建设川东北低空空域协调运行分中心。推进渠江航运枢纽建设，开工风洞子航运工程，加快构建区域内河水陆联运体系。充分发挥公路、铁路运输优势，与万州协作建设铁水联运港，开辟成都经达州至万州港进入长江的货运出海新通道。

（三）加快推进新型城镇化。围绕建设区域中心城市，坚持以人为本，完善规划，加快建设，强化管理，着力推进新型城镇化，力争全市户籍城镇化率提高0.7个百分点。

加强中心城区规划建设管理。坚持“三区”一体规划，根据区域中心城市体量规模和交通格局变化，在城市总体规划修编前期成果基础上，加快国土空间规划编制。提速建设城市骨干道路桥梁，扎实推进北城滨江和柳家坝、西城马踏洞和莲花湖、南城三里坪、小河嘴和长田坝等新区开发，新增建成区面积15平方公里、人口10万人。着力完善城市功能，抓好市政设施、公共服务设施建设，加快川鼓、404、老北站、野茅溪等片区棚改和莲花湖生态湿地公园、梨树坪湿地公园、城市运动公园、小河嘴海洋公园、城市应急备用水源建设，开工建设雷音铺公园，规划建设一批停车泊位、货车停车场。坚持建管并重，深入开展“城市综合管理年”活动，持续抓好“四城同创”，依法依规查处违法建设、违章搭建，开展广告乱设、车辆乱停、占道经营等突出问题专项整治，强化清扫保洁监督考核机制，抓好执法重心下沉和城市综合管理“点线面”示范，加强智慧城市建设，推动城市管理再上新台阶。

加强宜居宜业县城建设。以国土空间规划编制为契机，支持县城新区开发和产业新城建设，拓展城区发展空间，促进人口、资源、要素集聚，增强县城的承载力、吸纳力和带动力。引导各县加强城市综合管理，开展文明、卫生、园林、森林等城市创建，提升县城品质。支持大竹、渠县撤县设市，研究宣汉行政区划调整，引导宣汉、大竹、开江融入中心城区发展，引导渠县、万源借力中心城区发展。

加强重点镇特色镇建设。加强城镇基础设施和公共服务设施建设，做实产业、服务和居住功能，引导产业、人口向城镇集中，加快形成以县城为中心、以中心镇为重点的城镇群。结合资源特点、产业基础和发展潜力，因地制宜规划建设一批商贸重镇、工业强镇和旅游名镇。深入开展百镇建设行动，加快推进省级特色小镇、试点镇建设，力争新培育省级特色小镇1个、试点镇5个。

（四）大力推进乡村振兴和脱贫攻坚。农业农村是制约我市同步全面小康的最大短板。我们将扎实开展“精准脱贫决胜年”和“乡村振兴推进年”活动，抓重点、补短板，推动农业全面升级、农村全面进步、农民全面发展。

全力决胜脱贫攻坚。围绕“两不愁、三保障”，全力攻坚克难，确保万源、宣汉2个国贫县“摘帽”、49个贫困村退出、3.98万贫困人口脱贫。打好基础设施、产业培育、民生保障三大攻坚战，做好住房、产业、就业、教育等重点工作，加快摘帽县的乡“三有”[16]、贫困村“五有”[17]建设。加大政策、资金、项目倾斜力度，突出抓好13个深度贫困村脱贫退出。严格驻村工作队伍管理，强化贫困村“5+2”[18]和非贫困村“3+2”[19]帮扶，提高群众的认可度和满意度。持续开展“回头看”“回头帮”，确保已脱贫对象稳定脱贫。深入开展扶贫领域腐败和作风问题专项治理，为决胜脱贫攻坚提供坚强保证。

加快推进农业产业化。深化农业供给侧结构性改革，确保农业增加值增长3.5%。落实粮食安全生产责任，加强农田水利基本建设，新建高标准农田32.5万亩。坚持“抓二产带一产促三产”，做大做强本地农产品加工企业，持续抓好知名农产品加工企业招商，新引进全国知名农产品加工企业2家以上、新增农产品规上企业20家以上。加强特色优势产业基地建设，积极发展现代种养业，力争创建省级现代农业园区2个、市级5个。大力发展“互联网+农业”、“农业+旅游”、“农业+康养”等产业，扩大农产品贸易销售规模。突出富硒、绿色、生态等特色，加强品牌创建，抓好“巴山食荟”区域公用品牌的宣传和推广利用。

大力实施乡村振兴战略。以“五个振兴”为重点，编制完善达州乡村振兴规划，支持宣汉开展城乡融合综合改革试点，支持通川开展全省乡村振兴规划试点，打造乡村振兴“达州样板”。加强农村基础设施建设，新建农村公路1000公里，新增农村供水受益人口20万人。强力推进农村人居环境整治，深入实施村容村貌提升“六化”工程，重点抓好农村垃圾、污水、厕所治理“三大革命”。大力实施“三乡”计划，吸引能人回乡、支持业主兴乡、探索市民下乡，鼓励各类人才带着理念、资金和项目到农村创新创业。加强村民自治组织和平安乡村建设，推动村（社区）法律顾问全覆盖，促进乡村治理更加有序。全面推进农村集体产权制度、科技制度、要素保障机制等改革，激发乡村振兴活力。

（五）深入推进改革开放创新。我们将以更大的决心和更大的力度推进改革开放创新不断向纵深发展，厚植达州加快发展新优势，塑造经济增长新动能。

大力推进改革攻坚。深化供给侧结构性改革，加快煤炭、钢铁、水泥等重点行业重组整合和转型升级，抓好商业用房去库存，落实减税降费政策降成本，巩固“三去一降一补”成果。优化完善市区管理体制和城市管理执法体制，落实市区规划、建设、国土管理政策，理顺城管、食品药品监管等领域市区职责。深化机构改革，统筹推进城市、农村和食品药品“三大综合执法”重心下沉，提升监管实效。深化国资国企改革，明晰主业主责，做实资产，建立现代企业制度，推动市级平台公司“平台转型”和改革后的7类16户国企加快发展。深化要素市场化配置、投融资、财税金融、商事制度等改革，增强经济发展动能。鼓励基层根据改革发展实际，探索原创性、机制性和实用性改革创新。

努力扩大开放合作。贯彻落实省委“一干多支、五区协同”发展战略，坚持“四向拓展、全域开放”，主动融入全国全省开放大格局。加强重点产业招商，积极参与西博会、渝洽会、西（安）洽会和上海、广州进出口博览会等省内外重大投资促进活动，精心办好“中外知名企业达州行”、秦巴地区商品交易会等活动，深入开展“达商回引”工程，支持达商回达注册投资。加强与川东北各市和成都平原经济区规划对接、产业融合、设施互通、资源统筹、服务共享。深化与周边区域交通、物流、商贸等合作，推动达万协同发展，联动秦巴互惠发展，打造区域发展共同体。积极参与长江经济带、“一带一路”建设，扩大与京津冀、长三角、北部湾等地区交流合作，大力承接河北等省（市）产业转移。深化与浙江舟山扶贫协作，加快推进在物流、旅游、劳务等方面的合作。深化与央企省企、高等院校、科研院所、金融保险担保机构的合作，助推全市产业转型发展。支持企业拓展海外市场，推动“达州造”优势产品走出去。加强出口企业及产品培育，抓好经开区、大竹和宣汉园区等出口基地建设，确保进出口总额完成8200万美元。

加快推进创新发展。激励企业加大研发投入，力争研发经费支出占GDP比重达0.4%。围绕智能制造、微玻纤、玄武岩纤维、锂钾资源综合开发利用等重点产业，组建技术创新中心、产业技术研究院，推动资源就地深度开发。鼓励企业开展制度创新、管理创新、技术创新、模式创新，支持企业与高校、科研院所合作建设创新平台。加强重大科技成果转化，新增高新技术企业3家以上，确保科技对经济增长的贡献率达57%。大力开展“双创”行动，鼓励科技人员、青年、大学生创新创业，支持农民工返乡创业，新增市场主体1.4万个。

（六）切实加强生态环境建设。良好的生态环境，既是宝贵的发展资源，也是基本民生。我们将深入开展“环境治理巩固年”活动，确保全市生态环境质量不断改善。

持续抓好生态环境整治。强力推进中央和省环保督察及“回头看”反馈问题整改，加快实现销号清零。持续开展城乡环境综合治理，狠抓成达铁路沿线和主城区重要交通出入口周边环境整治。持续打好污染防治“八大战役”[20]，切实解决黑臭水体、工业废气、道路扬尘、餐饮油烟、畜禽养殖污染等突出环境问题，确保空气质量持续改善。全面落实河（湖）长制，加强州河、渠江等重点流域综合治理和岸线保护，确保国控断面优良率、城市集中式饮用水源地水质达标率100%。突出抓好环保基础设施建设，着力推进环保产业园、垃圾焚烧发电等重大项目建设，集中力量建成一批城镇污水处理和城乡垃圾处理设施。建立污染防治攻坚重点区域、重点行业、重点企业清单，实施重点生态功能区产业准入负面清单制度，严厉打击环境违法行为。

着力加强生态保护与修复。认真落实主体功能区规划，严格执行生态保护红线，抓好天然林、生态公益林保护，加强自然保护区、森林公园、地质公园、湿地公园保护。加强松材线虫病防治，打好主城区生态屏障保卫战。深入实施绿化美化达州行动，统筹抓好城市公园、道路绿化、广场美化香化建设，抓好沿州河、巴河、渠江、宝石湖等生态廊道建设和新一轮退耕还林，新增城市绿地2000亩，完成营造林30万亩。推进废弃矿山复耕和裸露山体整治，加强地质灾害防治，治理水土流失280平方公里，筑牢长江上游生态屏障。

推动形成绿色生产生活方式。引导“1+7”园区推行企业循环式生产、产业循环式组合、园区循环式改造，加快发展清洁能源、节能环保装备等绿色产业，积极推进钢铁、火电、水泥等传统产业绿色化改造，促进生产方式绿色化。推动建立市场化、多元化生态保护补偿机制。完成环保机构监测执法垂直管理改革。倡导绿色生活方式，支持采购绿色环保产品，推广新能源汽车和清洁能源汽车，推动充电基础设施建设，开展公共机构节约能源资源和绿色家庭、绿色学校、绿色社区等示范创建。

（七）统筹做好财税金融工作。面对复杂的宏观经济形势和新的财税政策，做好今年财税金融工作尤为重要。我们将一手抓风险防范化解、一手抓经济民生服务保障，统筹做好财税金融工作。

扎实做好财政工作。抢抓国家宏观政策逆周期调节机遇，下任务、压责任，全力向上争取一般性转移支付、政府专项债券、各类专项资金，确保争取工作走在全省前列。把握市场机遇，统筹加强土地收储和出让，保障城市基础设施和公共服务设施建设资金需求。强化财政对行政事业单位国有经营性资产的管理，继续加强对融资工作的依法科学统筹管理。改革财政支持企业发展方式，统筹工业、服务业、农业、科技等发展资金，采取补要素、补要点等措施支持企业发展，提高财政资金使用效益。抓好分险基金整合做实。强化预算管理，全面实施绩效管理，优化支出结构，确保有限的财政资金用在刀刃上。严格政府债务管理，坚决遏制隐性债务增量，积极化解债务存量。

认真做好税收工作。认真落实国家各项减税降费措施，密切关注政策动态，超前做好应对工作。加强税源培植，落实好招商引资企业税收优惠，支持企业发展壮大。加强税源监管，充分发挥市级财经大数据平台功能，确保应收尽收。加强税收征管，大力推行电子发票，落实外地房地产和建设施工企业就地注册入统纳税措施，推动各大商业卖场和专业市场统一结算集中办税，防止税收流失，确保税收稳定增长。

努力抓好金融工作。加强金融工作统筹调度，提高金融服务实体经济能力。加强人才引进和业务培训，充实金融机构工作力量，提升政府系统领导金融工作的能力水平。推动达州银行、达州农商银行做优做强，持续降低不良贷款率，支持异地设立分支机构。优选专业化证券机构，加强对企业上市、直接融资专题辅导培育。探索资产证券化。大力实施特色农产品保险工作，推动省农业担保公司在达设立全资担保机构实现全域农业担保。推动市中小企业担保公司做大做强。持续改善金融生态，完善社会信用体系，综合运用法纪手段惩戒失信企业、法人和自然人。健全市县地方金融监管体系，压实属地风险处置责任。

（八）加快发展教育医疗事业。教育医疗涉及千家万户，事关区域中心城市核心竞争力。我们将树立与高铁赛跑的精神，集中精力集中财力，全力推动教育医疗扩总量提质量上台阶。

倾力办好人民教育。坚持以公办教育为主满足人民群众最广泛的基础性需求，以民办教育为有益补充满足社会多样化选择需要。认真实施主城区普惠制学前教育三年攻坚计划，新改扩建公办幼儿园15所。巩固义务教育均衡发展成果，抓好农村薄弱学校建设，新改扩建中小学校70所。加快推进达高中迁建和达州中学翠屏校区建设，完成主城区中职学校整合入园，将闲置资源移交属地办中小学和学前教育，着力化解主城区学校“大班额”。扎实推动全市“1+7”重点高中提质量上档次创品牌，适应高考综合改革需要，引领公办教育加快发展。支持达州嘉祥、成实外学校等扩大招生，推动达州东辰、天立、川师大附属实验学校等建成招生。规范发展各类教育培训。加快西南职业教育园区品牌化发展，推进区域职业教育高地和技能型人才培养基地建设，提高人力资源就业质量和水平，满足产业发展人才需求。深化与国内外名牌大学合作，支持四川文理学院开展学术交流、扩容发展应用型大学。支持达州职业技术学院向综合类高职院校转型发展，加快建设达州中医药职业学院和达州技师学院。

着力提升医疗水平。深化与国内外知名医院合作办医，发展特色专科和高端服务，推动与省骨科医院的合作，力争开工建设华西达州医院、奥美德国际医院，确保建成华西达州妇女儿童医院。加强重大疾病防控，深化公立医院综合改革，办好办强市中心医院、市中西结合医院和7个县级人民医院，提高医疗服务和保障水平。落实社会办医优惠政策，促进民营医疗机构健康发展。推进中医药大健康产业成链发展，打造特色医疗、美容保健、中医药养生等品牌，满足人民群众多层次多样化的康养保健需求。

与此同时，我们还将加强全民健身场地建设，让更多群众享受到运动的健康和快乐；着力推进文化惠民工程，办好第七届全国新农村文艺展演和农民丰收节；加强统计工作，抓好全国第四次经济普查；大力发展妇女儿童、青少年、老年人、残疾人等事业，推动社会全面进步。

（九）切实保障和改善民生。为政之道，民生为本。我们将深入开展“民生改善实效年”活动，努力让全市人民享有更多的获得感、幸福感和安全感。

努力做好民生工作。持续加大民生投入，确保一般公共预算民生支出比例不低于70%。全力办好全市25项民生实事、中心城区20件民生实事。坚持就业优先，统筹抓好高校毕业生、农民工、退役军人等就业工作，支持企业稳定就业岗位，开展就业帮助，城镇新增就业3.7万人。深入实施全民参保计划，推进法定人员全覆盖，推动异地就医联网直接结算，扩大医保药品报销范围。加强住房保障，改造危旧房2万套、农村危房0.9万户、农村土坯房3.3万户。加快养老服务体系建设，新增（改造）养老床位1500张。完善社会福利、社会救助等保障制度，提高城乡居民低保和特困人员供养水平。加强农民工服务保障。做好退役军人服务管理工作。

深入推进社会治理。利用国有闲置资产，支持街道社区阵地建设。加强物业管理，构建和谐小区。全面实施“七五普法”规划，深入推进司法所规范化建设。坚持发展新时代“枫桥经验”[21]，强化诉源治理和网格化服务管理。切实抓好安全生产，持续开展消防、城市地下管网等各种安全隐患大排查大整治，坚决遏制重特大安全事故。加强食品药品监管执法，推动主城区餐厨垃圾无害化处理全覆盖，打造区域放心消费环境。狠抓以信息化为支撑的立体化社会治安防控体系建设，纵深推进扫黑除恶专项斗争，切实加强禁毒防艾工作。加强应急能力建设，健全应急管理机制，抓好防汛抗旱和地质灾害防治工作。搞好国家安全教育，提升国防动员能力。做好双拥共建工作，巩固军政军民团结。

四、加强政府自身建设

人民满意是政府工作的根本目标。我们将树牢“四个意识”，坚定“四个自信”，坚决做到“两个维护”，加快政府职能转变，加强作风建设，努力建设人民满意的服务型政府。

（一）严格依法行政。坚决维护宪法法律权威，依法规范行使职权，自觉运用法治思维和法治方式推动工作。认真贯彻法治政府建设实施纲要，推进地方政府规章等制度性建设，切实抓好重大行政决策合法性审查，确保政府行为于法有据。主动接受市人大和市政协监督，广泛听取和吸纳各方意见。完善政务公开，规范行政执法，加强权力运行监督体系建设，始终让权力在法治轨道和阳光下运行。加强法律法规学习宣传，教育引导全体公务人员敬畏法纪、敬重人民。

（二）加强政务服务。完善政府机构改革，做好“三定”方案制定和工作衔接。深化“放管服”[22]改革，完善权力、责任和负面三张清单，规范“双随机、一公开”监管。推进“证照分离”[23]，实施“多证合一” [24]，推行市场主体简易注销。深化“互联网+政务服务”，推进公共资源交易监管体制机制创新和全流程电子化。开展政务服务对标行动，着力营造一流的营商环境。排除一切干扰，冲破一切阻力，坚决确保项目审批服务、“最多跑一次”等4项改革方案落实到位。

（三）持续改进作风。严格落实中央八项规定精神和省、市委相关规定及实施细则，重点整治“表态多调门高、行动少落实差、督导检查重痕轻绩”等问题，严防“四风”反弹。改进调查研究，精简会议文件，把更多的时间和精力用在想大事谋大事上，用在解决具体问题和推动工作落实上。严格执行工作责任制，扎实抓好“群众最不满意十件事”的整改落实，坚决整肃庸政懒政怠政行为，确保各项决策部署全面落实、政策措施全面落地。落实正向激励和容错纠错机制，给干事者鼓劲，为担当者撑腰，大力营造想干事、敢干事、干成事的浓厚氛围。

（四）提升能力水平。开展学习型政府创建，加强新理论新知识的学习培训，提升理论政策和专业化水平。遵循经济规律、社会规律、自然规律，遵守程序规定，提升科学决策能力。善于去粗取精、去伪存真、由此及彼、由表及里；善于抓住主要矛盾和矛盾的主要方面，突出重点，突破难点，提升研究解决实际问题的能力；善于分析形势、把握全局，抓住关键、谋定而动，不断探索新机制、研究新方法，提升驾驭复杂局面的能力。

（五）永葆清廉本色。严格落实全面从严治党要求，切实履行“一岗双责”，深入推进政府系统党风廉政建设和反腐败工作。加强重点领域、重要环节和关键岗位监管，构建统一高效的审计监督体系，管好用好公共资源、公共资金、国有资产。严控“三公”经费，按照不低于5%的幅度压减一般性经费支出。全力支持纪检监察机关履行职责，严惩群众身边的“微腐败”。加强廉政教育，打造清正廉洁的干部队伍，保持干净干事的良好形象。

各位代表！达州发展正处在持续向好、追赶发展的关键时期，时代赋予我们崭新的历史使命，让我们更加紧密地团结在以习近平同志为核心的党中央周围，在省委省政府和市委的坚强领导下，抢抓机遇，开拓进取，为加快建成四川东出北上综合交通枢纽、川渝陕结合部区域中心城市，奋力争创全省经济副中心作出新的更大贡献，以优异成绩迎接新中国成立70周年和达州建市20周年！
